# Energy metabolic state in hypothermically stored boar spermatozoa using a revised protocol for efficient ATP extraction

**DOI:** 10.1242/bio.017954

**Published:** 2016-09-09

**Authors:** Quynh Thu Nguyen, Ulrike Wallner, Marion Schmicke, Dagmar Waberski, Heiko Henning

**Affiliations:** 1 Unit for Reproductive Medicine of Clinics/Clinic for Pigs and Small Ruminants, University of Veterinary Medicine Hannover, Buenteweg 15, Hannover 30559, Germany; 2 Department of Animal Sciences, University of Göttingen, Albrecht-Thaer-Weg 3, Göttingen 37075, Germany; 3 Clinic for Cattle, Endocrinology Laboratory, University of Veterinary Medicine Hannover, Hannover, Bischofsholer Damm 15, Hannover 30173, Germany; 4 Department of Equine Sciences, Faculty of Veterinary Medicine, Utrecht University, Yalelaan 112, Utrecht 3584 CM, The Netherlands

**Keywords:** ATP, Energy charge, Spermatozoa

## Abstract

Mammalian spermatozoa utilize ATP as the energy source for key functions on the route to fertilization. ATP and its precursor nucleotides ADP and AMP are regularly investigated in sperm physiology studies, mostly by bioluminescence assays. Assay results vary widely, mainly due to different efficiencies in nucleotide extraction and prevention of their enzymatic degradation. Here, we describe a revised, validated protocol for efficient phosphatase inhibition and adenine nucleotide extraction resulting in consistently high ATP concentrations exceeding previously reported values for boar spermatozoa up to 20-fold. The revised assay is applicable for determining ATP concentrations and adenylate energy charge in extracts from fresh and frozen samples, thereby allowing simultaneous assessment of semen samples from long-term storage experiments. After validation, the assay was applied to liquid-preserved boar spermatozoa stored at 17°C and 5°C for 24 and 72 h. Cooling to 5°C, but not storage duration, reduced ATP concentration in spermatozoa (*P*<0.05), which was accompanied by the appearance of AMP and ADP in the preservation medium. ATP and energy charge were highly correlated to the proportion of membrane-intact spermatozoa, supporting the idea of nucleotides leaking through disrupted membranes in cold-shocked cells. The present assay allows highly standardized studies of energy metabolism in spermatozoa.

## Abstract (precis)

**Summary:** A revised protocol for efficient extraction of ATP from boar spermatozoa is presented that consistently yields high ATP contents and energy charge values from fresh and frozen samples.

## INTRODUCTION

ATP is the energy source for key functions of spermatozoa on the route to fertilization. Spermatozoa specifically utilize ATP in energy-dependent cellular activities such as motility (Mukai and Okuno, 2004), capacitation (Travis et al., 2001), hyperactivation, and acrosome reaction (reviewed in Du Plessis et al., 2015). Due to the essential role of ATP for maintenance and regulation of cellular function, the determination of ATP concentration is included in many sperm physiology studies. ATP content in spermatozoa is commonly determined by bioluminescence using the firefly luciferin-luciferase assay in a wide variety of species including fish (Perchec et al., 1995), boar (Long and Guthrie, 2006), bull (Guminska et al., 1997), domestic poultry (Wishart, 1982; Long and Guthrie, 2006), and human (Blerkom et al., 1995).

Protocols vary widely between reports and evidence for assay accuracy is often lacking. Studies on fresh and stored boar semen using variants of the bioluminescence method report variable ATP contents ranging between 5 and 152 pmol/10^6^ sperm (Long and Guthrie, 2006; Dziekońska and Strzeżek, 2011; Dziekońska et al., 2013). Ideally, an ATP assay should allow detection of all intracellular ATP. Maximal detection of ATP depends on efficient phosphatase inhibition to prevent ATP degradation and an effective ATP extraction step. The immediate use of a phosphatase inhibitor cocktail containing acidic and alkaline phosphatases as well as tyrosine protein phosphatases in aqueous solution prior to ATP release was found to increase the amount of detectable ATP in sperm samples of turkey, rooster and boar (Long and Guthrie, 2006). A simple method for extraction of ATP and other nucleotides is boiling of the sample in the presence or absence of deionized water or a boiling buffer (Ford and Leach, 1998; Yang et al., 2002; Yi et al., 2008). Other commonly used reagents for ATP extraction, e.g. perchloric acid or Tris-borate buffer have been suggested to interfere with the luciferin-luciferase reaction (Lyons et al., 1986; Yang et al., 2002).

In spermatology research, long-term preservation experiments for several days require storing of samples for later assessment, ideally without changing the measured ATP content. Reliable ATP values of frozen ATP extracts would allow the analysis of stored samples on the same assay run, thereby excluding inter-assay variation.

Only a few spermatology studies consider the concentration of the ATP precursor nucleotides ADP and AMP. The relative available concentrations of ATP, ADP and AMP allow the calculation of energy charge (EC) as first defined by Atkinson and Walton (1967). Surprisingly, little information is available in the literature about the EC of spermatozoa, although the EC is regarded as a more distinct indicator of the metabolic energy status of living cells than ATP concentrations alone (Du Toit et al., 1993).

The objective of the present study was to develop and validate a revised protocol for efficient nucleotide extraction and measurement of ATP levels and energy charge from diluted boar spermatozoa which allows frozen storage of the samples without compromising nucleotide concentrations. Protocol revision was based on the ATP and adenylate energy charge assay from Ford and Leach (1998) and key features of the ATP quantification assay described by Long and Guthrie (2006) for spermatozoa. The revised assay was applied to determine ATP content and energy charge in boar spermatozoa preserved at 17°C and 5°C with consideration of the nucleotide concentration in semen extender media.

## RESULTS

### Assay development

#### Experiment 1

**Fig. 1.**
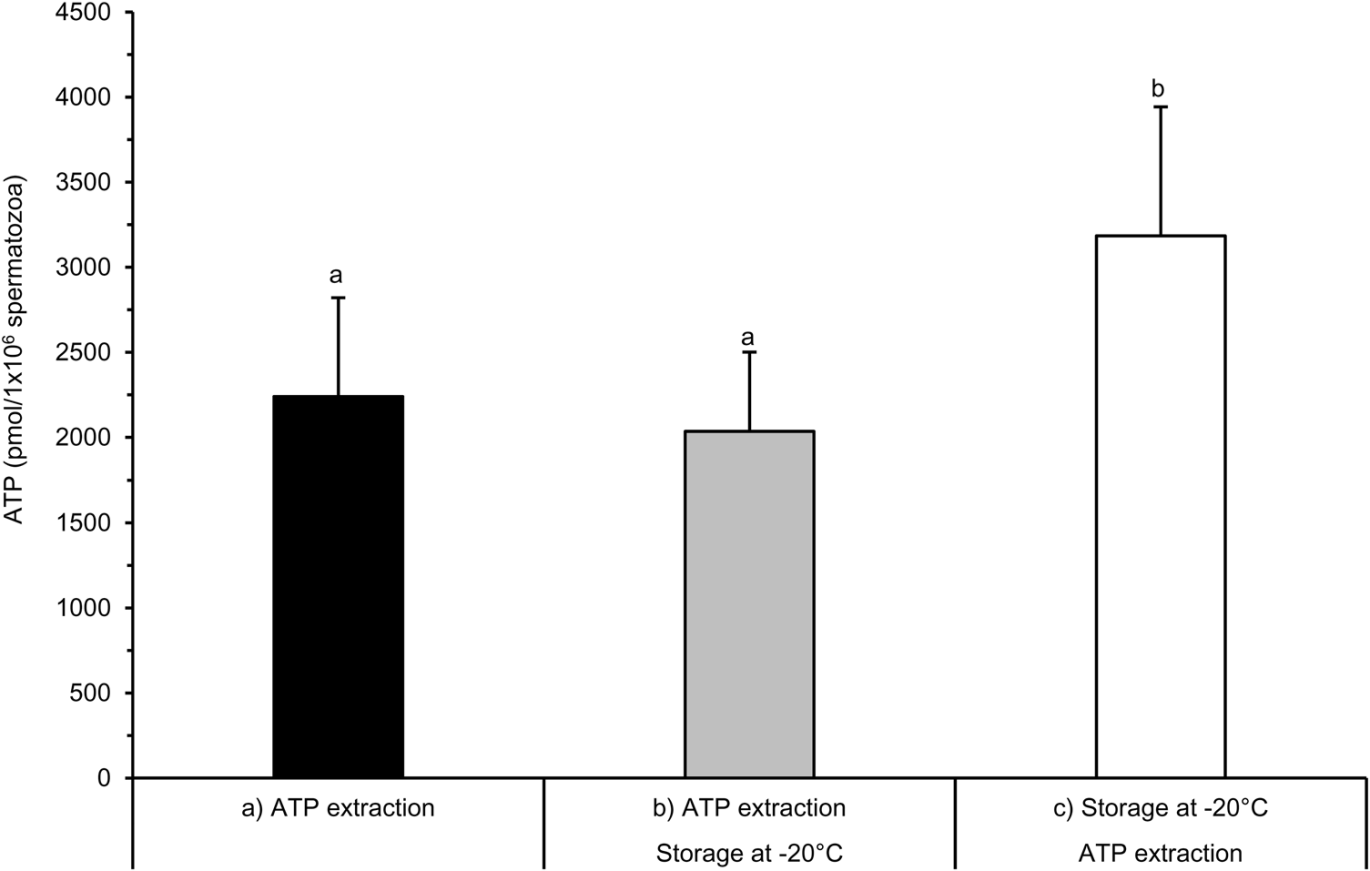
**Evaluation of ATP assay processing variants after phosphatase inhibition in diluted semen samples.** Immediate ATP extraction (A; control) was compared with frozen-storage of subsamples before (B) or after (C) ATP extraction. Subsequent ATP determination was performed by the luciferin-luciferase reaction. Values are mean±s.d.; *n*=11 semen samples. Different superscripts (a, b) indicate significant differences between treatments (*P*<0.05, Wilcoxon signed-rank test).

Levels of ATP in boar spermatozoa were similar when ATP was immediately measured after phosphatase inhibition and ATP extraction (control treatment A: 2240 pmol ATP/10^6^ spermatozoa) or from samples stored frozen after phosphatase inhibition and ATP extraction (treatment B: 2035 pmol ATP/10^6^ spermatozoa; Fig. 1). Unexpectedly, when samples were stored frozen after phosphatase inhibition and then thawed for ATP extraction, higher ATP contents (treatment C: 3185 pmol ATP/10^6^ spermatozoa) were noted (*P*<0.05).

#### Experiment 2

**Fig. 2.**
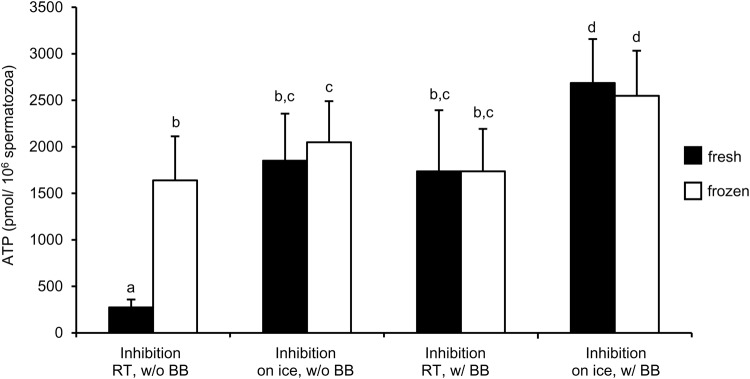
**Comparison of different processing conditions for determination of ATP concentration by the luciferin-luciferase reaction in diluted boar semen samples.** Phosphatase inhibition was either performed at room temperature (RT) or on ice. An aliquot of each sample was immediately further processed and analysed (fresh) while the other part was stored at −20°C (frozen) before ATP extraction. ATP extraction in samples was performed with or without a boiling buffer (BB). Different lower case superscripts (a-d) indicate significant differences between samples (fresh or frozen) for different treatments and between fresh and frozen samples. Values are mean± s.d.; *n*=6 boars; *P*<0.05, Student's *t*-test for paired observations.

**Fig. 3.**
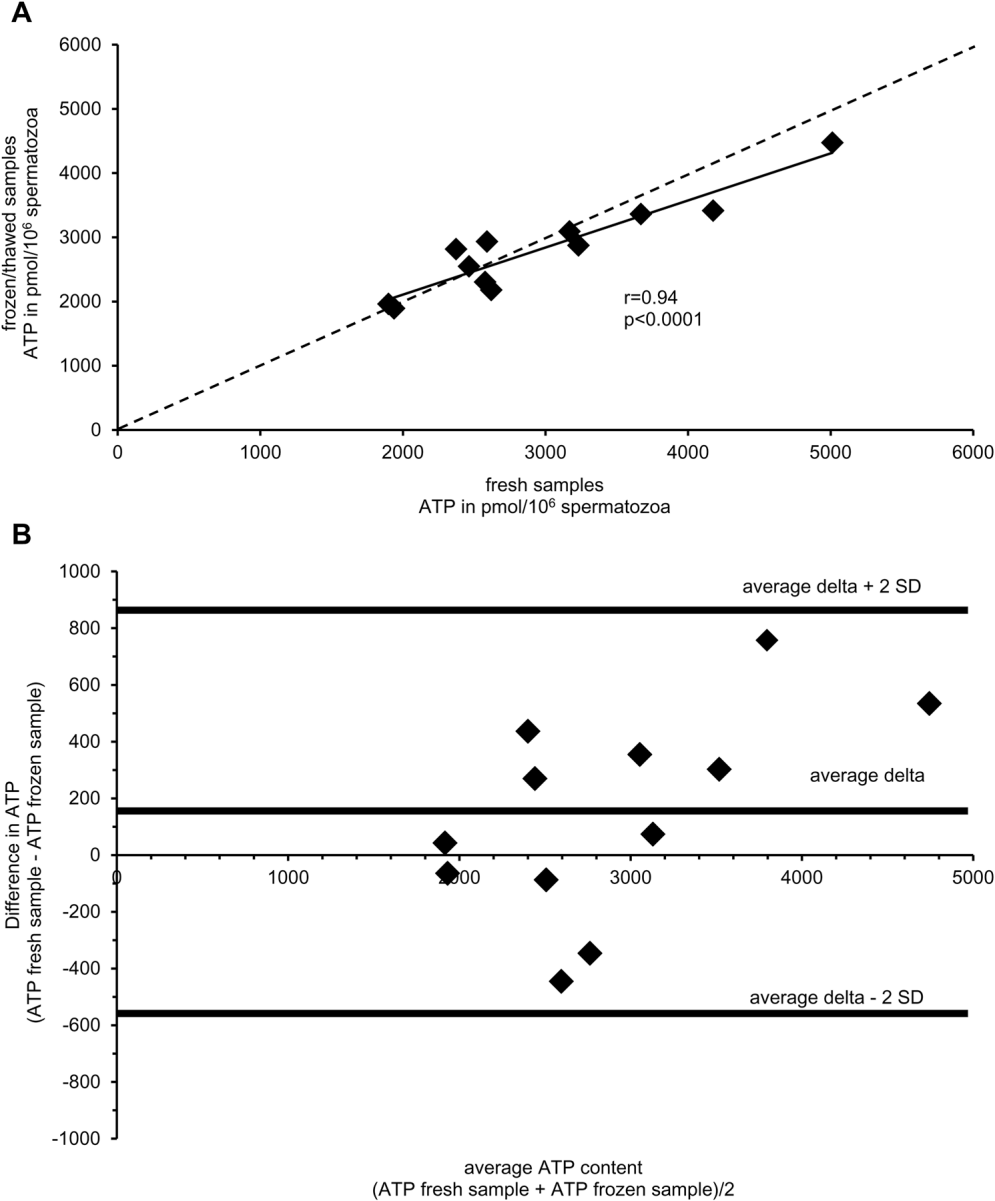
**Comparison of ATP content from fresh analysed and frozen-stored samples.** (A) Correlation between the ATP concentration in subsamples of diluted boar semen after phosphatase inhibition and either immediate ATP extraction (fresh) or ATP extraction after storage at −20°C (frozen; *n*=12 semen samples; Pearson's correlation coefficient). For all samples, phosphatase inhibition was done on ice with a boiling buffer. The dashed line indicates the equation where *x*=*y*. (B) Bland–Altman plot to evaluate agreement between results from fresh and frozen samples (*n*=12 semen samples).

Both inhibitor treatments – on ice for 30 min or the use of a boiling buffer – increased the amount of extracted ATP, when ATP extraction was done immediately after phosphatase inhibition (fresh samples, *P*<0.05; Fig. 2). A higher ATP content in extracts from fresh and frozen samples was detected when inhibitor treatment took place on ice (*P*<0.05). The combined use of phosphatase inhibitor treatment on ice and use of a boiling buffer resulted in the highest values of detectable ATP (*P*<0.05). No difference in freshly analysed and frozen samples was detected when either inhibitor treatment on ice or a boiling buffer or both were used for sample preparation and ATP extraction (*P*>0.05). ATP content of samples analysed fresh or stored frozen after phosphatase inhibition correlated significantly (r=0.94, *P*<0.01; Fig. 3A). The mean difference between ATP extracts of fresh and frozen samples was 153 pmol/10^6^ sperm, suggesting that fresh analysed samples may contain occasionally more ATP than after freezing and thawing (Fig. 3B). However, a systematic decrease could not be detected.

### Assay validation

#### Experiment 3

##### Intra-assay and inter-assay variation for ATP assay and energy charge assay

After determining optimum conditions for effective nucleotide extraction from boar spermatozoa, the procedure was tested for repeatability, i.e. the intra-assay variation for ATP assessments and energy charge assessments. The coefficients of variation were on average 5.7% (range: 3.0–9.1%) for the ATP assay (Table S1A) and on average 4.5% (range: 2.4–7.0%) for the adenylate energy charge assay (Table S1B). The inter-assay variation for the ATP assay was on average 8.0% (range: 3.6–12.3%; Table S2A), and for the adenylate energy charge assay on average: 3.4% (range: 1.7–4.9%; Table S2B). Freezing of the samples had no impact on ATP content and results of the adenylate energy charge assay (Table S2A,B).

### Assay application

#### Experiment 4

##### ATP and energy charge in spermatozoa of hypothermic stored semen samples

**Fig. 4.**
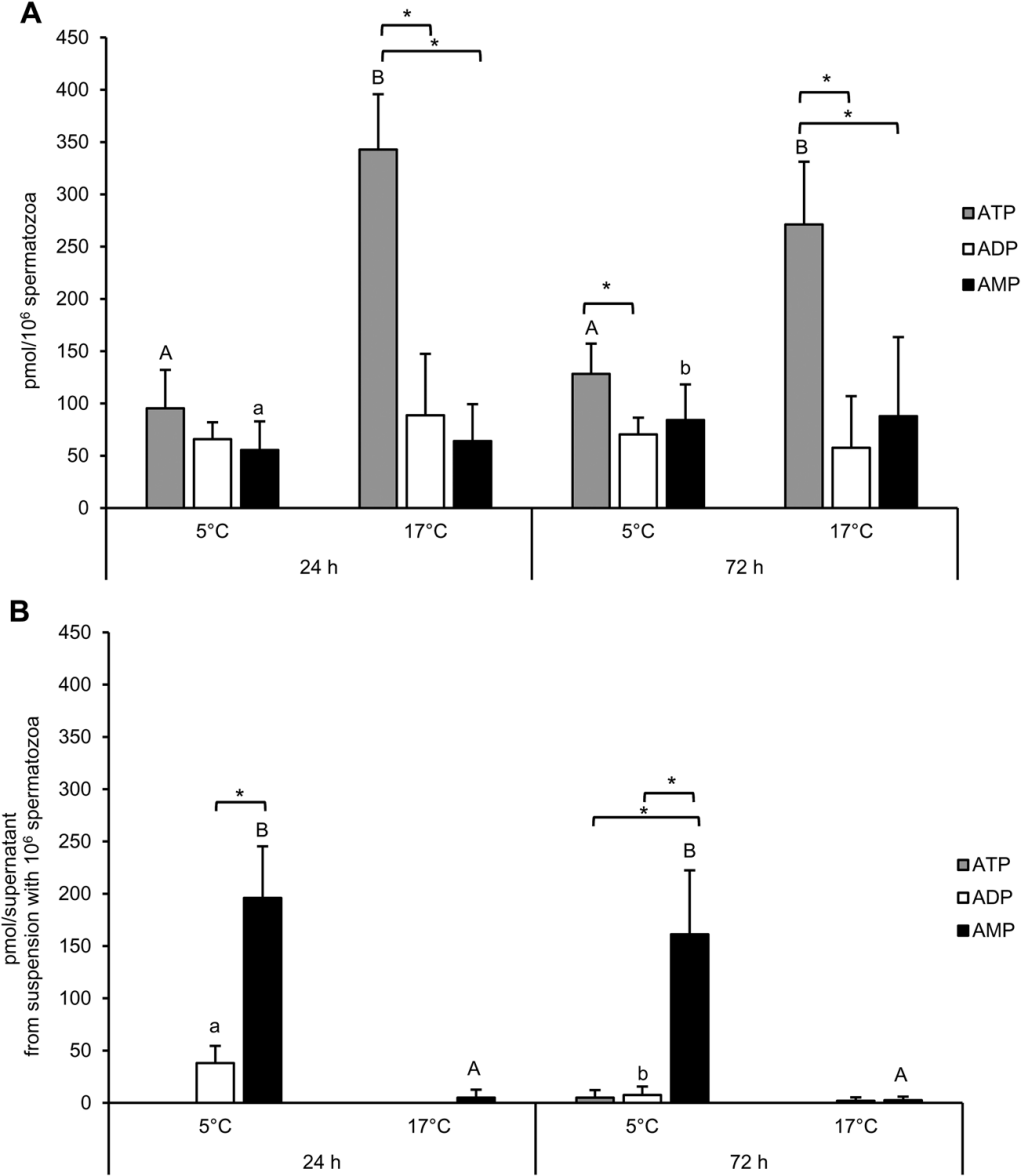
**ATP, ADP and AMP content of boar spermatozoa and medium after storage of semen in BTS-extender.** Diluted boar semen was stored at 5°C and 17°C (*n*=6 boars) for 24 h and 72 h. Spermatozoa (A) and supernatant (B) were separated by centrifugation through a discontinuous Percoll^®^ gradient before assessment of nucleotides. All values are mean±s.d. Different lower case superscripts (a, b) indicate significant differences in the concentration of a given nucleotide between storage times (Student's *t*-test for paired observations; *P*<0.05). Different uppercase superscripts (A,B) indicate significant differences in the concentration of a given nucleotide between storage temperatures at a given storage time (Student's *t*-test for paired observations; *P*<0.05). An asterisk indicates significant differences in ATP, ADP, and AMP concentration within a sample at a given storage time and temperature (Student's *t*-test for paired observations; *P*<0.05).

The ATP concentration was higher in spermatozoa stored at 17°C compared to 5°C (*P*<0.05; Fig. 4A). Storage time had no impact on the ATP and ADP concentration. The AMP concentration increased from 24 h until 72 h of storage in spermatozoa held at 5°C. The ATP concentration and energy charge, but not ADP and AMP concentration, in spermatozoa were highly correlated with the percentage of spermatozoa with intact plasma and acrosomal membranes (Table 1).

**Table 1.**
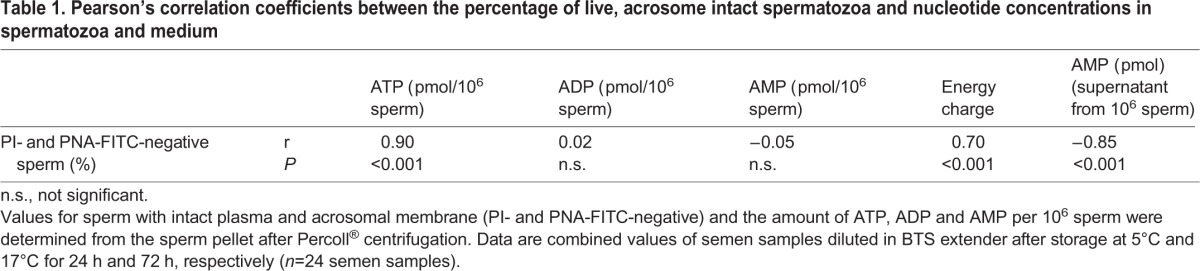
**Pearson’s correlation coefficients between the percentage of live, acrosome intact spermatozoa and nucleotide concentrations in spermatozoa and medium**

The ATP content of the isolated semen extender and residual seminal plasma was negligible irrespective of storage time and temperature (Fig. 4B). Almost no ADP and AMP were detectable in the supernatant of samples stored at 17°C. On the contrary, a considerable amount of ADP (58% of the amount found in spermatozoa) was detected in the supernatant of semen samples stored for 24 h at 5°C. The ADP concentration decreased until 72 h of storage. The AMP concentration in the supernatant of semen samples at 5°C was more than threefold higher than the AMP concentration of spermatozoa at 24 h and more than twofold higher after 72 h storage. The concentration of AMP in the supernatant was inversely correlated with the percentage of spermatozoa with intact plasma and acrosomal membranes (Table 1).

There was no ATP and almost no ADP and AMP detected in seminal plasma directly after semen collection (Table 2). The energy charge of fresh ejaculated spermatozoa ranged between 0.85 and 0.92.

**Table 2.**
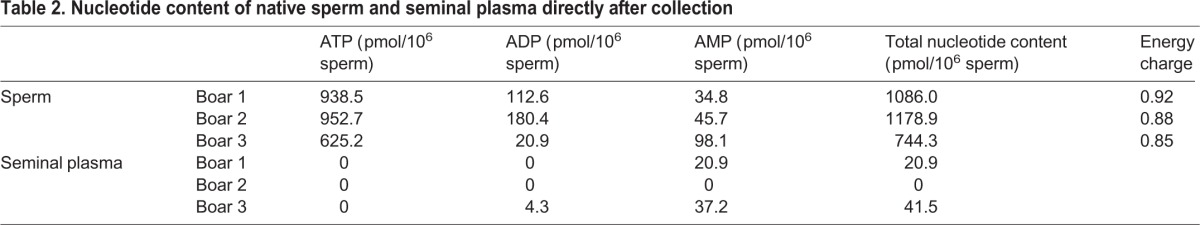
**Nucleotide content of native sperm and seminal plasma directly after collection**

## DISCUSSION

In this study, a revised protocol for efficient extraction of ATP for subsequent measurement of cellular ATP and energy charge in boar spermatozoa is presented using a commercial luciferin-luciferase reagent. With the method reported here consistently 10- to 20-fold higher ATP concentrations in boar spermatozoa were measured compared to previous reports (Long and Guthrie, 2006; Dziekońska and Strzeżek, 2011; Dziekońska et al., 2013). A key feature of the revised assay is an improved method for ATP extraction by phosphatase inhibition on ice and use of a boiling buffer. Efficient and consistent ATP extraction is considered as the most critical step for intracellular ATP measure. A reliable method for extraction of adenine nucleotide from intact cells is determined by complete release of intracellular adenine nucleotides, complete and irreversible inactivation of all adenine nucleotide converting enzymes, and no interference with enzymes used in the luciferin-luciferase assay (Lundin and Thore, 1975). The improved method therefore enhanced sensitivity and could be used for samples in which only low numbers of spermatozoa are present. Attempts to modify ATP extraction were triggered by the observation that freezing of samples before ATP extraction revealed higher ATP concentrations compared to fresh semen or samples frozen after ATP extraction. It is assumed that freezing induced membrane disruption and thereby gave access to previously unreleased ATP residues. Our observation is different to results from the study of Long and Guthrie (2006), but might be explained by the different semen preparations used for comparison of processing variants. Long and Guthrie (2006) used the sperm-rich fraction of a native ejaculate, while we used a full ejaculate that was diluted with a commercial semen extender (Beltsville Thawing Solution; BTS). High concentrations of cations in native ejaculates, especially Zn^2+^ and Cu^2+^ (Li et al., 2015; Lopez Rodriguez et al., 2013; Strzezek et al., 1995; Lavon and Boursnell, 1975) may have a dose-dependent quenching effect on emitted light in an ATP assay (Wen et al., 2001) and thus could impair the sensitivity of the system. BTS extender contains 3.4 mM EDTA (Johnson et al., 2000) and may have buffered free cation concentrations to low levels. Furthermore, direct effects of EDTA on light production in an ATP assay are dose-dependent (Wen et al., 2001). In addition, EDTA acts as inhibitor of ATP-dependent metalloproteases and thereby may contribute to preventing ATP degradation.

We observed that phosphatase inhibition on ice as described by Guthrie et al. (2011) in striped bass spermatozoa and the use of a Tricine boiling buffer (Ford and Leach, 1998) yielded highest ATP concentration regardless whether ATP was extracted from fresh or frozen samples. Therefore, ice conditions effectively prevented ATP degradation during phosphatase inhibitor treatment. In the absence of ice, degradation processes lead to a loss of 40% detectable ATP within 15 min (Long and Guthrie, 2006). After the inhibition step, heating the sample in the presence of a boiling buffer was performed to extract ATP. Previously, the Tricine buffer as used in the present study was shown to be the most effective buffer among the ten tested for ATP extraction and did not interfere with the luciferin-luciferase system (Webster et al., 1980). Since then the Tricine buffer has been regularly used in sperm ATP-assays of different species (Ho and Suarez, 2003; Yi et al., 2008).

The present assay revealed a linear relationship between ATP standard concentration and relative light units (RLU) from 31 to 2000 pmol ATP. This contrasts with the report of Long and Guthrie (2006) that values higher than 160 pmol ATP required a log/log transformation to achieve linearity. It is important to note that sensitivity and precision of the luciferin-luciferase assay is high (Holm-Hansen and Karl, 1978; Long and Guthrie, 2006). Consequently, sources of variation are present in the efficiency of ATP extraction and prevention of ATP degradation rather than in the luciferin-luciferase reaction. High correlations between ATP concentration in fresh and frozen-stored samples together with low intra-assay variation for both ATP and EC values demonstrate that the present method is suitable for routine assessment of cellular energy metabolism in stored samples. Application of this assay for measurement of ATP concentrations and energy charge in extracts from fresh and frozen aliquots from the same semen samples was highly repeatable with low intra- and inter-assay variation. In order to minimize assay variation, we recommend performing the phosphatase inhibition (on ice with a boiling buffer) and freezing of all samples of an experimental unit at −20°C. Later, extraction of ATP from frozen-thawed samples should be performed on one day and all samples that need to be compared should be analysed in the same luciferin-luciferase assay run. EC in native boar semen was high level (0.9) and corresponds to EC measured in freshly ejaculated boar spermatozoa after determination of adenine nucleotides by a fluorometric enzymatic assay (Kamp et al., 2003). Similarly high EC levels (0.8-0.9) are considered as physiological in freshly ejaculated human spermatozoa (Chulavatnatol and Haesungchatern, 1977). It is important to note that the presence of spermatozoa with altered membranes may influence ATP and EC values in semen samples. Leakage of adenine nucleotide through disrupted membranes into the surrounding medium may result in low ATP/ADP/AMP concentration in a sub-population of cells with defect membranes. In fact, the present study revealed a high positive correlation between the proportion of membrane-intact sperm and ATP and EC levels, respectively, whereas AMP in the supernatant was negatively correlated to the energy measures. Consequently, in samples with distinct amounts of membrane damaged cells, ATP and EC values are expected to reflect the proportion of viable (membrane intact) cells in the sample rather than the energy status of living cells. Any determination of energy status of spermatozoa therefore should include information on the integrity of the plasma membranes. In accordance with observations of Long and Guthrie (2006), the ATP content of seminal plasma was negligible in raw semen samples. In the present study membrane disruption was induced by lowering the storage temperature of the semen to 5°C. Under these conditions when boar spermatozoa were cooled below lipid phase transition temperatures (between 30°C and 10°C; Drobnis et al., 1993; Schmid et al., 2013), increased ADP and AMP concentration were found in the extender medium regardless of the storage period. The hypothesis that cooling-induced rearrangement of lipid domains increases membrane permeability (Drobnis et al., 1993) and thus leads to leakage of intracellular nucleotides through disrupted membranes was confirmed. The lower intracellular ATP concentrations in samples stored at 5°C may partially result from loss of the precursor nucleotides AMP and ADP and partially from impaired activity of ATP-generating enzymes.

In conclusion, a revised protocol for an efficient and highly repeatable ATP extraction in boar spermatozoa is presented which allows freezing of samples at −20°C prior to extraction without affecting the ATP content and energy charge. The revised ATP assay is suitable for studies of the energy metabolism in boar spermatozoa, particularly when only low sperm numbers are available or samples need to be stored for later assessment. In any case, cell membrane integrity of the original semen sample should be considered to avoid misleading data interpretation.

## MATERIALS AND METHODS

### Reagents

Unless otherwise stated, chemicals were obtained from Sigma-Aldrich (Steinheim, Germany), Merck (Darmstadt, Germany), and Roth (Karlsruhe, Germany).

### Animals and semen collection

Semen was collected from a total of six healthy, mature and fertile boars (*sus scrofa domesticus* Linnaeus, 1758) housed at the Unit for Reproductive Medicine of Clinics, University of Veterinary Medicine Hannover. The boars (age range: 1.5 to 5 years) belonged to the breed Piétrain, German Large White or were crossbred animals. Housing and management of the animals complied with the national laws and guidelines. The ejaculates were collected by the gloved hand method into disposable semen collection bags with integrated filter (Minitüb GmbH Tiefenbach, Germany), which were enclosed in insulated plastic thermos cups pre-heated to 38°C. Immediately after collection, semen was transferred to the laboratory and isothermically (33°C) diluted with Beltsville Thawing Solution (BTS; Minitüb). Sperm concentration was assessed by the ‘Thoma neu’ counting chamber with phase-contrast microscope (Zeiss, Jena, Germany) at 400× magnification. Only normospermic ejaculates were used for the experiments i.e. ejaculates with ≥100 ml volume, ≥160×10^6^ sperm ml^−1^ concentration, ≥70% motile spermatozoa, ≤25% morphological abnormal sperm. Diluted semen with a final concentration of 20×10^6^ sperm ml^−1^ was kept at room temperature and used on the day of collection to develop and validate the ATP and EC assay. In experiments utilizing diluted semen (Experiment 4), 100 ml samples were kept for 90 min at room temperature. Then, samples were transferred to a storage unit (17°C). Cooling to 5°C was achieved by holding semen for 60 min at 17°C, followed by 60 min at 10°C before samples were stored at 5°C.

### ATP assay

The ATP assay included the following steps:

1. Preparation of an ATP standard curve
2. Phosphatase inhibition
3. ATP extraction
4. ATP detection with luciferin/luciferase reaction.

Variations of the single steps were tested as described in the respective experiment section.

### Assay development

#### Experiment 1: Sample storage after phosphatase inhibition

Experiment 1 was based on the protocol of Long and Guthrie (2006) and had the aim to test two methods which have been suggested as having optimal processing steps to store samples in a frozen state for later ATP assessment without affecting the ATP content. In the control procedure, 100 µl diluted semen samples were treated with 1 µl phosphatase inhibitor cocktail (P5726, Sigma-Aldrich) at room temperature (RT) for 30 min. Thereafter, ATP was extracted from the samples and subsequently analysed with a commercial firefly luciferin-luciferase assay (Sigma-Aldrich). In the two test treatments, either the supernatant after ATP extraction was stored for three days at −20°C before thawing and used in the ATP assay or, the semen sample was frozen after treatment with phosphatase inhibitor cocktail and extraction of ATP was accomplished at post-thawing. ATP extraction was always performed without the use of a boiling buffer. The experiment was carried out twice. One sample was lost during processing, resulting a total number of 11 samples.

#### Experiment 2: Phosphatase inhibition and ATP extraction

**Fig. 5.**
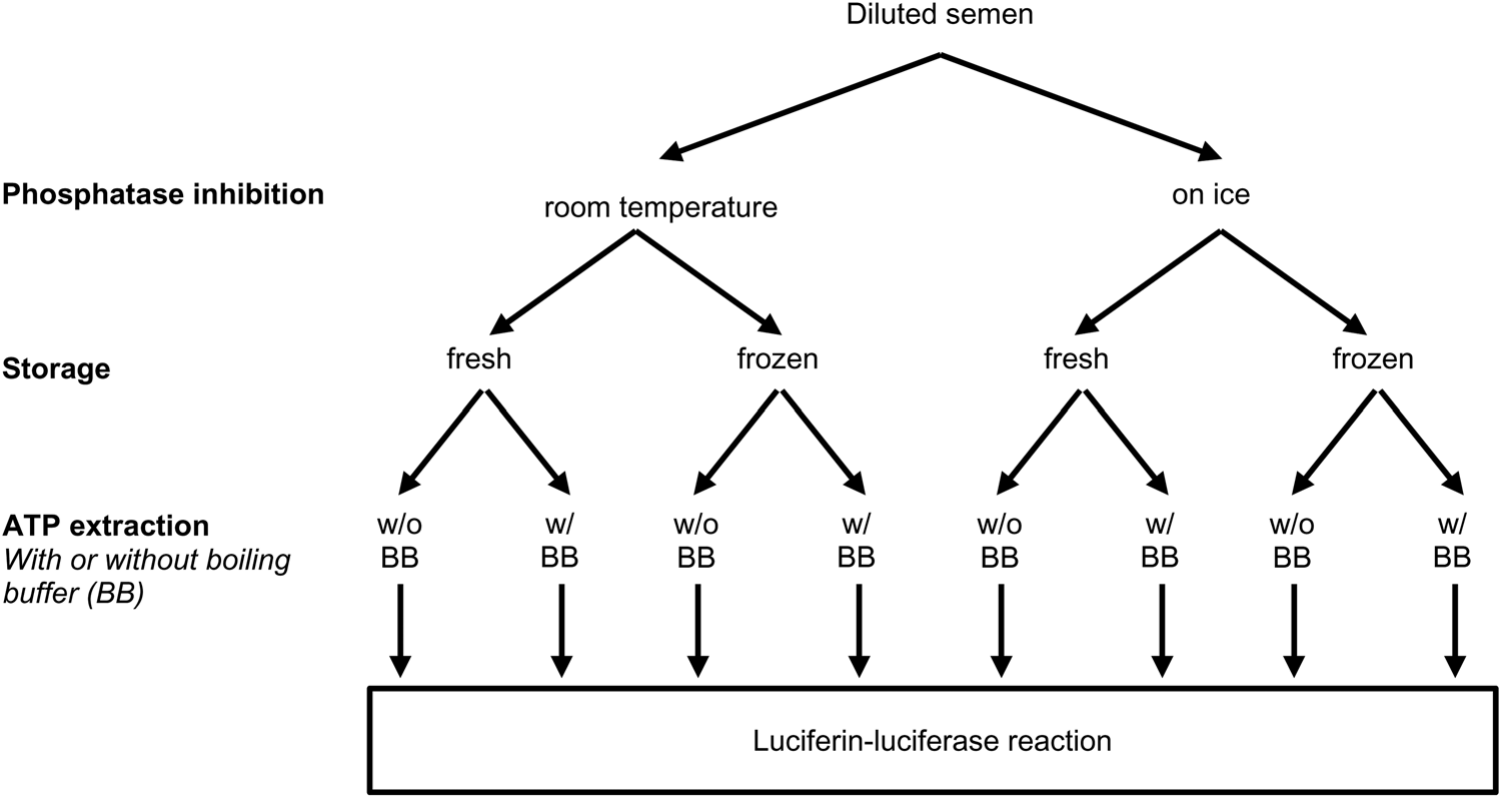
**Flow chart illustrating the design of Experiment 2.** A full description of all steps is given in the Materials and Methods section.

Results from Experiment 1 indicated that ATP extraction from samples treated with inhibitor at room temperature is suboptimal and that a small volume of the supernatant after ATP extraction is inconvenient for further processing. Therefore, Experiment 2 focussed on optimizing the ATP extraction procedure. A 2×2-factorial experiment was designed (Fig. 5) to evaluate firstly how the temperature during a 30 min phosphatase inhibitor treatment (RT or on ice) and the use of a boiling buffer during ATP extraction would affect the amount of extractable ATP and secondly, whether the amount of extractable ATP differs between directly processed samples and frozen/thawed aliquots after three days of storage at −20°C.

### Assay validation

#### Experiment 3: Intra-assay and inter-assay variation for ATP and energy charge assay

The optimum conditions for effective ATP extraction from boar spermatozoa required a phosphatase inhibitor treatment on ice for 30 min before sample freezing. A boiling buffer was used during nucleotide extraction (c.f. Experiment 2). Using these conditions, the repeatability of ATP and energy charge assessments was determined. Intra-assay variation was assessed by calculating the coefficient of variation of six different assay runs from aliquots of the same diluted semen samples. Inter-assay variation was determined by comparing results of aliquots from fresh and frozen/thawed samples at different days.

##### ATP standard curve preparation

An ATP standard solution was prepared by dissolving the content (1 mg) of one vial (2×10^6^ µmol) of ATP standard (FLAAS, Sigma Aldrich, St Louis, MO, USA) with 1 ml AMPUWA water (Fresenius Kabi, Bad Homburg, Germany). From this stock solution a serial dilution with concentrations of 62.5, 125, 250, 500, 1000, 2000 pmol ml^−1^ was prepared. A standard dilution series was prepared for each day. Aliquots of the stock solution were stored at −20°C until use.

A volume of 25 µl of ATP standard concentrations (62.5, 125, 250, 500, 1000, and 2000 pmol ATP ml^−1^) and a blank sample (AMPUWA water) were added to the wells of a 96-well microtiter plate with white walls and clear bottoms (Greiner Bio-One, Frickenhausen, Germany). Then, the ATP assay mix solution (FLAAM, Sigma-Aldrich) was diluted 1:25 with dilution buffer (FLAAB, Sigma-Aldrich). A volume of 100 µl of the diluted ATP assay mix was added to each well by using an automatic pipette (Biohit eLine E 1000, Biohit, Rosbach, Germany). The plate was briskly swirled and the amount of produced light immediately measured with a Tecan GENios Pro plate reader (Tecan Group Ltd., Männedorf, Switzerland) controlled by Magellan software (Version V5.03, Tecan Group Ltd., Männedorf, Switzerland). All standards and blank samples were prepared and measured in duplicate.

**Fig. 6.**
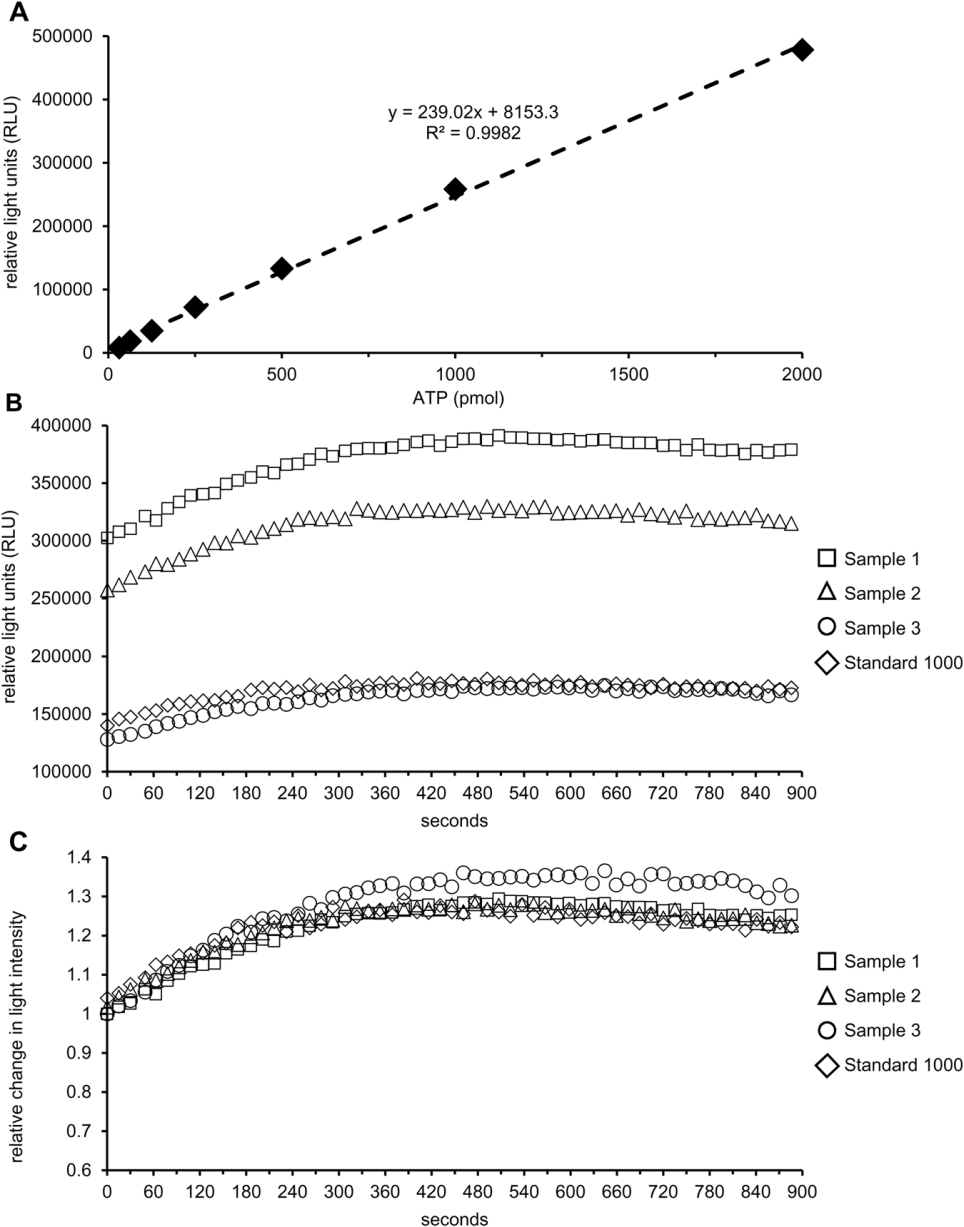
**ATP standard curve and changes in light emission over time for ATP assay.** (A) Example of linearity of standard curves with the linear range from 31.25 pmol to 2000 pmol ATP. (B) Relative light emission of samples or standard solutions increases over a time irrespective of the initial starting values. (C) Relative changes in light intensity over time are similar for samples or standard solutions (e.g. 1000 pmol ATP).

The RLU obtained from the blank sample were subtracted from the light units measured for each ATP standard concentration. The corrected values for the RLU are proportional to the amount of ATP in the standard samples. A linear regression between RLU and ATP concentrations was established (c.f. Fig. 6A).

##### Phosphatase inhibition and ATP extraction

Unless otherwise indicated, 100 µl of a semen sample were mixed with 1 µl phosphatase inhibitor cocktail (P5726, Sigma-Aldrich) and kept for 30 min at room temperature (RT) or on ice. After inhibitor treatment, fresh or frozen samples were boiled either with or without a boiling buffer solution (50 mM Tricine, 10 mM MgSO_4_, 2 mM EDTA, pH 7.8) for nucleotide extraction. Samples without addition of boiling buffer were directly heated for 10 min in a Thermomixer 5436 (Eppendorf, Hamburg, Germany). When a boiling buffer was used, tubes containing 900 µl boiling buffer were heated for 5 min at 95°C before the samples were added. After addition of samples, the mixture was heated for 10 min at 95°C. Thereafter, the tubes were chilled on ice for 10 min and then centrifuged at 5000×***g*** for 30 min at 4°C (Universal 30 RF, Hettich, Tuttlingen, Germany). The supernatant was used for determination of ATP and EC.

##### ATP detection with luciferin-luciferase reaction

ATP was measured using a commercial firefly luciferin-luciferase assay (FL-AA, Sigma-Aldrich). A volume of 25 µl of each sample was added to a well of a 96-well microtiter plate. Then, 100 µl of the diluted ATP reaction mix were added by an automatic pipette. The plate was swirled briskly and the amount of light produced was immediately measured. Each assay run included a ‘blank’ sample and ATP standard sample. All samples were prepared and analysed in duplicate. After correction of all RLU values for background light as assessed by the blank sample, the standard sample was used to calculate a correction factor for each assay run. Light production in samples and ATP standards increases over time and any delay between start of the reaction and reading the emitted light may lead to a bias in the data (Fig. 6B,C). The correction factor for each assay run was calculated by dividing the light units for the standard sample in a given assay run by the light units obtained during preparation of the standard curve for a given ATP concentration. The correction factor was applied to calculate the corrected value of relative light units for each sample. Relative light units were averaged for each sample and the ATP concentration determined by using the linear equation of the ATP standard curve (*y*=*a*×*x*+*b*), where *y* is RLU, *x* is the ATP concentration, *a* is slope and *b* is y-intercept.

##### Energy charge measurement

The energy charge was determined by modification of procedures described by Ford and Leach (1998). The modifications are described below. Three aliquots (100 µl each) of the samples to be analysed for nucleotides were each incubated with 25 µl of one of three different buffers (Buffer A, B and C). Reaction buffer A was used for determination of ATP and contained 75 mM Tricine, 5 mM MgCl_2_ and 0.0125 mM KCl (pH 7.5). Reaction buffer B was used for determining the combined amount of ATP and ADP. In addition to reaction buffer A, buffer B contained 0.1 mM phosphoenolpyruvate (P7002, Sigma-Aldrich) and 0.08 µg µl^−1^ of pyruvate kinase (P1506, Sigma-Aldrich). Phosphoenolpyruvate and pyruvate kinase stock solutions were centrifuged for 5 min at 10,000×***g***, the pellet diluted 1:3 in 20 mM Tris and 0.1% bovine serum albumin (A2153, Sigma-Aldrich; pH 7.5) before addition to Buffer B.

Phosphoenolpyruvat (PEP) reacts with ADP to form ATP. The reaction is catalysed by pyruvate kinase (Eqn 1).
(1)
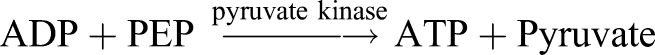

Tubes containing reaction buffer A and B were incubated at 30°C for 30 min.

Reaction buffer C was used for determining the combined amount of ATP, ADP, and AMP. In addition to reaction Buffer B, Buffer C contained 0.1 µg µl^−1^ of adenylate (myo) kinase (Sigma-Aldrich, M 3003). The adenylate (myo) kinase stock solution was centrifuged for 5 min at 10,000×***g***, the pellet diluted 1:12 in 20 mM Tris and 0.1% bovine serum albumin, pH 7.5) before addition to Buffer C. The adenylate kinase converts AMP in the sample to ADP (Eqn 2) and subsequently phosphoenolpyruvate and pyruvate kinase convert ADP to ATP (Eqn 1).
(2)
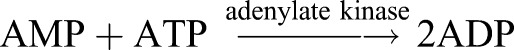

Tubes containing reaction buffer C were incubated at 30°C for 90 min. All three tubes were boiled at 95°C for 3 min to stop reactions and then chilled on ice until assayed for ATP content. Measurement and calculation of the ATP content was similar to the procedure described for the ATP assay. Concentrations of ADP and AMP were obtained by subtracting the results from the appropriate measurements. The energy charge was calculated as described by Ball and Atkinson (1975; Eqn 3).

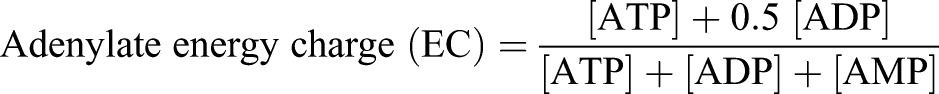



### Assay application

#### Experiment 4: ATP and energy charge in spermatozoa of hypothermic stored semen samples

Boar spermatozoa are usually preserved in the liquid state at 17°C. During processing and storage, molecules from membrane-damaged spermatozoa may diffuse from the intracellular compartment to the medium. The presence of nucleotides in the liquid phase may be a confounding factor for the determination of ATP content and energy charge in diluted spermatozoa. Diluted semen samples (*n*=6) were stored at 17°C and 5°C for 24 h and 72 h. At each time of storage, extender and spermatozoa were separated by centrifugation of samples through a discontinuous Percoll^®^ gradient with layers of 70% and 35% Percoll^®^ working solution. Working solutions of Percoll^®^ were prepared as detailed previously (Henning et al., 2015). While spermatozoa are enriched in the pellet of the tube, semen extender stays on top of the 35% Percoll^®^ layer. The concentration of nucleotides for calculation of the adenylate energy charge (ATP, ADP and AMP) determined from the pelleted spermatozoa and from the extender. In addition, the percentage of sperm with intact plasma and acrosome membranes was determined of pelleted sperm.

After ejaculation, spermatozoa are mixed with the secretions of the accessory sex glands. Semen and seminal plasma from three of the six boars were assayed for nucleotide concentrations after isothermic (36°C) separation on a discontinuous Percoll^®^.

##### Percoll^®^ centrifugation

Aliquots of 4 ml extended semen stored at 5 and 17°C or ejaculated semen were carefully layered over the two-step Percoll^®^ gradients (35% and 70%), and tubes were centrifuged at 300×***g*** for 10 min followed by 15 min at 750×***g*** without stopping the centrifuge. After centrifugation, the supernatants were centrifuged again for 3 min at 3360×***g*** and checked microscopically to be free of spermatozoa and collected for ATP and energy charge measurement. The pelleted spermatozoa were gently resuspended in BTS extender to a final concentration of 20×10^6^ sperm ml^−1^ for assessment of the percentage of sperm with intact plasma and acrosome membranes, and measurements of ATP and energy charge.

##### Assessment of plasma and acrosome membrane integrity

Integrity of plasma and acrosomal membranes was assessed using combined staining with propidium iodide (PI), FITC-PNA, and Hoechst 33342, respectively. Briefly, aliquots of 5 µl sample of diluted semen after storage at 5 and 17°C or from pellets after Percoll^®^ centrifugation were mixed with 980 μl pre-warmed HEPES-buffered saline solution (HBS; 137 mM NaCl, 20 mM HEPES, 10 mM glucose, 2.5 mM KOH, 1 mg ml^−1^ BSA, pH 7.4 at 20°C, 300±5 mOsmol kg^−1^), 5 μl PI stock solution (1 mg ml^−1^), 5 μl FITC-PNA stock solution (600 μg ml^−1^) and 5 μl Hoechst 33342 stock solution (150 μg ml^−1^) and incubated for 5 min at 38°C in an incubator. Flow cytometric analysis of stained samples was performed on a DAKO Galaxy flow cytometer (Dako Cytomation GmbH, Hamburg, Germany), equipped with a 488 nm blue argon laser (20 mW) and an HBO-lamp for excitation of the dyes. The HBO excitation spectrum was restricted with filters to wavelengths between 270 nm and 405 nm (main peak: 365 nm). The cytometer was equipped with filters for green (BP 537.5/22.5 nm; FITC), red (LP 630 nm; PI) and blue (BP 465 nm; Hoechst 33342) fluorescence, respectively. The sperm population was identified by characteristic forward and side scatter distribution patterns of Hoechst 33342 positive, i.e. DNA-containing, events. Fluorescence intensities (in logarithmic mode) were collected for 10,000 events per sample, at a rate of 400 to 800 events s^−1^. Data were analysed using FloMax software (Partec GmbH, Münster, Germany). Spectral overlap was compensated post acquisition.

### Statistical analysis

Data were analysed using Excel (Microsoft Office 2007, Microsoft Corporation, Washington, United States) and Statistic Analysis Software (SAS, Version 9.2, Cary, NC, USA). The sample size was *n*=6 boars, unless otherwise stated. Data were tested for normal distribution with a Shapiro–Wilk test. Logarithmic transformation was applied to nucleotide concentrations in supernatant from semen extender (Experiment 4) to achieve normal distribution. The Student's *t*-test for paired observations was used to compare treatments with normally distributed data (Experiment 2, Experiment 4). If data could not be transformed into a normal distribution, comparisons were performed using the Wilcoxon's signed-rank test (Experiment 1). Measuring agreement between subsamples processed fresh and frozen after phosphatase inhibition was evaluated by Bland–Altman plot (results for semen samples from Experiment 2 and Experiment 3 (inter-assay variation) were combined; *n*=12). Data from semen samples in Experiment 4 were pooled and Pearson's correlation coefficient was calculated for selected parameters (*n*=24). All data in this study are reported as mean±standard deviation (s.d.). The significance level was set at *P*<0.05.

## References

[BIO017954C1] Atkinson D. E. and Walton G. M. (1967). Adenosine triphosphate conservation in metabolic regulation. Rat liver citrate cleavage enzyme. *J. Biol. Chem.* 242, 3239-3241. 6027798

[BIO017954C2] Ball W. J. Jr and Atkinson D. E. (1975). Adenylate energy charge in Saccharomyces cerevisiae during starvation. *J. Bacteriol.* 121, 975-982. 1090610 10.1128/jb.121.3.975-982.1975 PMC246026

[BIO017954C3] Blerkom J. V., Davis P. W. and John L. (1995). ATP content of human oocytes and developmental potential and outcome after *in-vitro* fertilization and embryo transfer. *Hum. Reprod.* 10, 415-424. 7769073 10.1093/oxfordjournals.humrep.a135954

[BIO017954C4] Chulavatnatol M. and Haesungchatern A. (1977). Stabilization of adenylate energy charge and its relation to human sperm motility. *J. Biol. Chem.* 252, 8088-8091. 21188

[BIO017954C5] Drobnis E. Z., Crowe L. M., Berger T., Anchordoguy T. J., Overstreet J. W. and Crowe J. H. (1993). Cold shock damage is due to lipid phase transitions in cell membranes: a demonstration using sperm as a model. *J. Exp. Zool.* 265, 432-437. 10.1002/jez.1402650413 8463792

[BIO017954C6] Du Plessis S. S., Agarwal A., Mohanty G. and Van Der Linde M. (2015). Oxidative phosphorylation versus glycolysis: what fuel do spermatozoa use? *Asian J. Androl.* 17, 230-235. 10.4103/1008-682X.135123 25475660 PMC4650467

[BIO017954C7] Du Toit D., Bornman M. S., Van Der Merwe M. P., Du Plessis D. J. and Oosthuizen J. M. C. (1993). Differential sperm motility scoring and sperm ATP concentrations. *Andrology* 30, 69-71. 10.3109/01485019308988371 8420507

[BIO017954C8] Dziekońska A. and Strzeżek J. (2011). Boar variability affects sperm metabolism activity in liquid stored semen at 5°. *Pol. J. Vet. Sci.* 14, 21-27. 10.2478/v10181-011-0003-1 21528707

[BIO017954C9] Dziekońska A., Fraser L., Majewska A., Lecewicz M., Zasiadczyk L. and Kordan W. (2013). Effect of commercial long-term extenders on metabolic activity and membrane integrity of boar spermatozoa stored at 17°C. *Pol. J. Vet. Sci.* 16, 517-525. 10.2478/pjvs-2013-0072 24195287

[BIO017954C10] Ford S. R. and Leach F. R. (1998). Bioluminescent assay of the adenylate energy charge. *Method. Mol. Biol.* 102, 69-81. 10.1385/0-89603-520-4:69 9680610

[BIO017954C11] Guminska M., Kedryna T., Laszczka A., Godlewski M., Slawinski J., Fabianczyk B. S., Kwiecinska T., Rajfur Z. and Wierzuchowska D. (1997). Changes of ATP level and iron- induced ultra- weak photon emission in bull spermatozoa, caused by membrane peroxidation during thermal stress. *Quarterly* 2, 131-138. 9241365

[BIO017954C12] Guthrie H. D., Welch G. R., Theisen D. D. and Woods L. C. III (2011). Effects of hypothermic storage on intracellular calcium, reactive oxygen species formation, mitochondrial function, motility, and plasma membrane integrity in striped bass (Morone saxatilis) sperm. *Theriogenology* 75, 951-961. 10.1016/j.theriogenology.2010.10.037 21247623

[BIO017954C13] Henning H. Ngo T. T. and Waberski D. (2015). Centrifugation stress reduces the responsiveness of spermatozoa to a capacitation stimulus in in vitro-aged semen. *Andrology* 3, 834-842. 10.1111/andr.12064 26226856

[BIO017954C14] Ho H.-C. and Suarez S. S. (2003). Characterization of the intracellular calcium store at the base of the sperm flagellum that regulates hyperactivated motility. *Biol. Reprod.* 68, 1590-1596. 10.1095/biolreprod.102.011320 12606347

[BIO017954C15] Holm-Hansen O. and Karl D. M. (1978). Biomass and adenylate energy charge determination in microbial cell extracts and environmental samples. *Methods Enzymol.* 57, 73-85. 10.1016/0076-6879(78)57010-9

[BIO017954C16] Johnson L. A., Weitze K. F., Fiser P. and Maxwell W. M. C. (2000). Storage of boar semen. *Anim. Reprod. Sci.* 62, 143-172. 10.1016/S0378-4320(00)00157-3 10924823

[BIO017954C17] Kamp G., Busselmann G., Jones N., Wiesner B. and Lauterwein J. (2003). Energy metabolism and intracellular pH in boar spermatozoa. *Reproduction* 126, 517-525. 10.1530/rep.0.1260517 14525534

[BIO017954C18] Lavon U. and Boursnell J. C. (1975). The split ejaculate of the boar: contributions of the epididymides and seminal vesicles. *J. Reprod. Fert.* 42, 541-552. 10.1530/jrf.0.0420541 1123818

[BIO017954C19] Li Y., Wang A., Taya K. and Li C. M. (2015). Declining semen quality and steadying seminal plasma ions in heat-stressed boar model. *Reprod. Med. Biol.* 14, 171-177. 10.1007/s12522-015-0205-9 PMC5715830 29259414

[BIO017954C20] Long J. A. and Guthrie H. D. (2006). Validation of a rapid, large-scale assay to quantify ATP concentration in spermatozoa. *Theriogenology* 65, 1620-1630. 10.1016/j.theriogenology.2005.06.020 16364417

[BIO017954C21] Lopez Rodriguez A., Rijsselaere T., Beek J., Vyt P., Van Soom A. and Maes D. (2013). Boar seminal plasma components and their relation with semen quality. *Syst. Biol. Reprod. Med.* 59, 5-12. 10.3109/19396368.2012.725120 23083319

[BIO017954C22] Lundin A. and Thore A. (1975). Comparison of methods for extraction of bacterial adenine nucleotides determined by firefly assay. *Appl. Microbiol.* 30, 713-721. 812422 10.1128/am.30.5.713-721.1975 PMC187260

[BIO017954C23] Lyons G., Bilgeri Y. R., Zanzinger A., Berzin M. and Mendelsohn D. (1986). Extraction and estimation of ATP from human spermatozoa. *Andrologia* 18, 455-460. 10.1111/j.1439-0272.1986.tb01808.x 3800003

[BIO017954C24] Mukai C. and Okuno M. (2004). Glycolysis plays a major role for adenosine triphosphate supplementation in mouse sperm flagellar movement. *Biol. Reprod.* 71, 540-547. 10.1095/biolreprod.103.026054 15084484

[BIO017954C25] Perchec G., Jeulin C., Cosson J., Andre F. and Billard R. (1995). Relationship between sperm ATP content and motility of carp spermatozoa. *Cell Sci.* 108, 747-753. 10.1242/jcs.108.2.747 7769016

[BIO017954C26] Schmid S., Henning H., Oldenhof H., Wolkers W. F., Petrunkina A. M. and Waberski D. (2013). The specific response to capacitating stimuli is a sensitive indicator of chilling injury in hypothermically stored boar spermatozoa. *Andrology* 1, 376-386. 10.1111/j.2047-2927.2013.00045.x 23427145

[BIO017954C27] Strzezek J., Torska J., Borkowski K., Glogowski J., Wysocki P. and Holody D. (1995). The Biochemical characteristics of boar seminal plasma during high ejaculation frequency. *Reprod. Dom. Anim.* 30, 77-84. 10.1111/j.1439-0531.1995.tb00608.x

[BIO017954C28] Travis A. J., Jorgez C. J., Merdiushew T., Jones B. H., Dess D. M., Diaz-Cueto L., Storey B. T., Kopf G. S. and Moss S. B. (2001). Functional relationships between capacitation dependent cell signalling and compartmentalized metabolic pathways in murine spermatozoa. *J. Biol. Chem.* 276, 7630-7636. 10.1074/jbc.M006217200 11115497

[BIO017954C29] Webster J. J., Chang J. C., Manley E. R., Spivey H. O. and Leach F. R. (1980). Buffer effects on ATP analysis by firefly luciferase. *Analyt. Biochem.* 106, 7-11. 10.1016/0003-2697(80)90111-6 7191217

[BIO017954C30] Wen G., Paul Voroney R., Schoenau J. J., Yamamoto T. and Chikushi J. (2001). Assessment of ionic quenching on soil ATP bioluminescence reaction. *Soil Biol. Biochem.* 33, 1-7. 10.1016/S0038-0717(00)00104-8

[BIO017954C31] Wishart G. J. (1982). Maintenance of ATP concentrations in and of fertilizing ability of fowl and turkey spermatozoa in vitro. *J. Reprod. Fert.* 66, 457-462. 10.1530/jrf.0.0660457 7175802

[BIO017954C32] Yang N.-C., Ho W.-M., Chen Y.-H. and Hu M.-L. (2002). A convenient one step extraction of cellular ATP using boiling water for luciferin- luciferase assay of ATP. *Anal. Biochem.* 306, 323-327. 10.1006/abio.2002.5698 12123672

[BIO017954C33] Yi Y. J., Li Z. H., Kim E. S., Song E. S., Kim H. B., Cong P. Q., Lee J. M. and Park C. S. (2008). Comparison of motility, acrosome, viability and ATP of boar sperm with or without cold shock, resistance in liquid semen at 17°C and 4°C, and frozen- thawed semen. *Asian Aust. J. Anim. Sci.* 21, 190-197. 10.5713/ajas.2008.70351

